# The atypical sphingolipid SPB 18:1(14Z);O2 is a biomarker for *DEGS1* related hypomyelinating leukodystrophy

**DOI:** 10.1016/j.jlr.2023.100464

**Published:** 2023-10-27

**Authors:** Andreas J. Hülsmeier, Sandra P. Toelle, Peter Bellstedt, Christian Wentzel, Angela Bahr, Konstantinos Kolokotronis, Thorsten Hornemann

**Affiliations:** 1 Institute of Clinical Chemistry, University Hospital Zurich, University of Zurich, Zurich, Switzerland; 2 Department of Pediatric Neurology, University Children's Hospital, Zurich, University of Zurich, Zurich, Switzerland; 3 Department of Women's and Children's Health, Pediatric Oncological and Neurological Research, Uppsala University, Uppsala, Sweden; 4 Institute of Medical Genetics, University of Zurich, Schlieren, Zurich, Switzerland

**Keywords:** Sphingolipids, DEGS1, hypomyelinating leukodystrophy, sphingolipidosis, HLD18, biomarker, SPT, FADS3, ceramide

## Abstract

Sphingolipids (SL) represent a structurally diverse class of lipids that are central to cellular physiology and neuronal development and function. Defects in the sphingolipid metabolism are typically associated with nervous system disorders. The C4-dihydroceramide desaturase (*DEGS1*) catalyzes the conversion of dihydroceramide to ceramide, the final step in the SL *de-novo* synthesis. Loss of function mutations in *DEGS1* cause a hypomyelinating leukodystrophy, which is associated with increased plasma dihydrosphingolipids (dhSL) and with the formation of an atypical SPB 18:1(14Z);O2 metabolite. Here, we characterize two novel *DEGS1* variants of unknown significance (VUS), provide a structural model with a predicted substrate binding site, and propose a regulatory link between DEGS1 and fatty acid desaturase 3 (FADS3). Both VUS involve single amino acid substitutions near the C-terminus within conserved regions of the enzyme. Patient 1 (p.R311K variant) shows severe progressive tetraspasticity, intellectual disability, and epilepsy in combination with brain magnetic resonance imaging (MRI) findings, typical for DEGS1-related leukodystrophy. Patient 2 (p.G270E variant) presents with delayed psychomotor development, oculomotor apraxia, and a normal brain MRI. Plasma from the p.R311K carrier showed a significantly elevated dhSL species and the presence of SPB 18:1(14Z);O2, while the plasma SL profile for the p.G270E variant was not altered. This suggests the p.R331K variant is pathogenic, while the p.G270E appears benign. As an increase in dihydroSL species is also seen in other pathological disorders of the SL metabolism, the SPB 18:1(14Z);O2 seems to be a more specific biomarker to discriminate between pathogenic and benign *DEGS1* variants.

## Graphical Abstract

**Figure:**
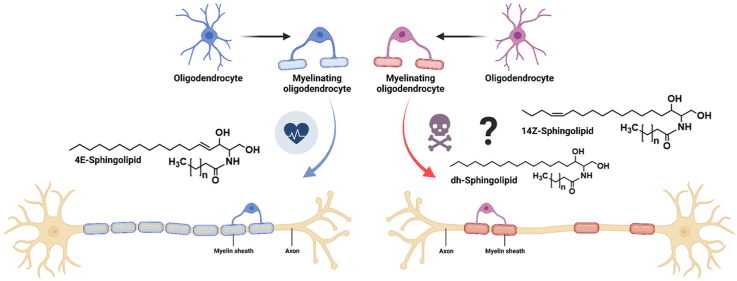

Hypomyelinating leukodystrophies are genetic disorders characterized by a primary lack of myelin deposition, resulting in an aberrant white matter formation of the brain (1). Mutations in *DEGS1* have been described in the context of hypomyelinating leukodystrophy 18 (HLD18) (2, 3, 4). Clinically, affected patients present with severe progressive tetraspasticity, variable degrees of intellectual disability, no verbal communication, and epilepsy in most cases.

Sphingolipids (SL) are integral parts of cellular membranes and essential components of neuronal tissues. Mutations in genes encoding for enzymes involved in the de novo biosynthesis and the degradation of SL lead to the accumulation of specific enzyme substrates or toxic lipid metabolites. Such changes in sphingolipid profiles may serve as potential biomarkers for diagnosing and predicting neurological disorders. Common neurodegenerative disorders like Alzheimer's disease (AD), Parkinson's disease (PD), amyotrophic lateral sclerosis (ALS), and Huntington's disease have been linked to altered sphingolipid levels and metabolism (5, 6, 7, 8, 9, 10, 11, 12, 13).

Ceramides (Cer) are the central metabolites in the SL metabolism and the precursor for complex SLs, such as sphingomyelins and glycosphingolipids. Increased Cer has been implicated with cell death, cellular stress responses, senescence, and cell cycle arrest (14). Cer *de-novo* synthesis starts in the ER, typically with the condensation of serine and palmitoyl-CoA by the serine palmitoyl transferase (SPT). In addition, SPT can also metabolize acyl-CoAs with chain lengths in the range from C14 to C18, forming SLs with straight and branched sphingoid bases (SPB) (15). Subsequently, the de novo formed SPB is *N*-acylated by a group of ceramide synthases (CerS) forming dihydroceramide (dhCer) and finally converted to Ceramide (Cer) by the insertion of a Δ4,5-*trans* (Δ4E) double bond. This last reaction is catalyzed by the Δ4-dihydroceramide desaturase (DEGS1) (16, 17). The cellular level of dhCer is maintained at a significantly lower concentration compared to Cer. Both dhCer and Cer can be transported to the Golgi apparatus by sphingolipids transfer proteins, such as ceramide transfer protein (CERT), four-phosphate adaptor protein 2 (FAPP2), and ceramide-1-phosphate transfer protein (CPTP), leading to the formation of distinct classes of complex sphingolipids, such as sphingomyelin, glycosphingolipids or ceramide-1-phosphate (18).

DEGS1 plays a crucial role in differentiating dihydrosphingolipids from sphingolipids primarily by converting dhCer to Cer. In vitro characterization of DEGS activity using rat liver microsomes showed the highest activity towards dhCer, whereas the activity was reduced towards dihydro-sphingomyelin and absent with dihydro-glucosylceramide (19). Loss of function mutations in *DEGS1* lead to an increase of dhCer species, which are also converted to more complex sphingolipids, such as dihydro-sphingomyelins and dihydro-glycosphingolipids. In addition, DEGS1 deficiency results in the formation of an atypical sphingolipid metabolite (SPB 18:1(14Z);O2), which is formed in a metabolic bypass reaction, independent of DEGS1 activity by the fatty acid desaturase 3 (FADS3) (14, 15). Under physiological conditions, FADS3 catalyzes the formation of a second Δ14,15-*cis* (Δ14Z) double bond in the SPB backbone of Cer, predominately downstream of DEGS1 activity, forming the double unsaturated SAdienine (20) (Fig. 1).

**Fig. 1:**
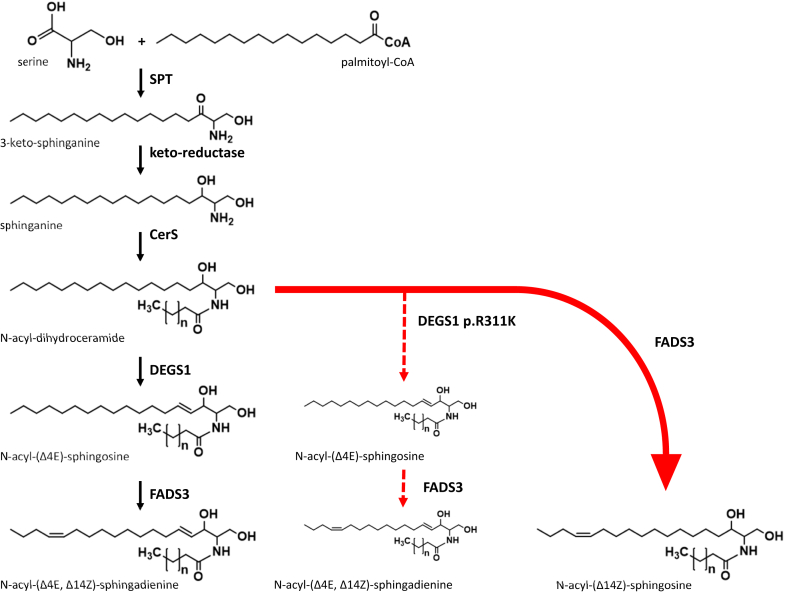
Ceramide de novo biosynthesis typically starts with the condensation of serine and palmitoyl-CoA by serine palmitoyl transferase (SPT) to form 3-ketosphinganine, which is reduced to sphinganine by keto-reductase. Ceramide synthases (CerS) N-acylate sphinganine to form dihydroceramide. Δ4-dihydroceramide desaturase (DEGS1) introduces the Δ4E double bond to form N-acyl-(Δ4E)-sphingosine (ceramide) from dihydroceramide. The formation of N-acyl-(Δ4E, Δ14Z)-sphingosine is catalyzed by fatty acid desaturase 3 (FADS3) that inserts the Δ14Z double bond to ceramide. N-acyl-(Δ14Z)-sphingosine (SPB 18:1(14Z);O2-based ceramide) is formed in a bypass reaction of FADS3 in the event of DEGS1 deficiency (red arrow).

## Materials and Methods

The study protocol was approved by the Ethics Committee of the University Hospital Zurich and all patients were provided with written, informed consent. All experiments were performed in accordance with the guidelines and regulations of the Ethics Committee of the University Hospital Zurich and abide by the Declaration of Helsinki principles.

### Genetic testing

DNA was extracted from blood samples of the examined family members (index patient and parents) and trio exome sequencing was performed. The amplification of the exome was performed using the xGen® Exome Research Panel v1.0 (IDT). After paired-end sequencing (NovaSeq 6000 S1 Reagent Kit (300 cycles), 150 fwd-150 rev, Q30-value: 92.9) on a NovaSeq 6000 sequencer (Illumina Inc.), the coding regions of the currently known genes as well as the exon-intron-boundaries have been analyzed up to 6 base pairs into the intron (alignment: NextGene V2.4.2.3 (Softgenetics); analysis: NextGene Viewer V2.4.2.3 (Softgenetics). DEGS1 variant: coverage 133x, variant allele frequency 100%.

### Long-chain base analyses

SL extraction from plasma was performed as described previously (15). Methanol, 500 μl, containing the internal standards, 200 pmol d7-sphinganine, and 200 pmol d7-sphingosine was added to 50 μl plasma. The extraction was maintained for 1 h at 37°C with agitation at 1400 rpm in a Thermomixer (Eppendorf, Germany). Proteins were precipitated by centrifugation for 5 min at 16,000 *g*. 500 μl of the supernatant was collected, and HCl was added to yield a final concentration of 1.5 M. Lipids were hydrolyzed for 16 h at 65°C. The reaction was neutralized by adding 100 μl of 10M KOH, and the released long-chain bases were extracted in 625 μl chloroform while maintaining alkaline conditions with ammonia. The organic phase was washed twice with 0.05% aqueous ammonia. Hydrolyzed lipids were dried and resuspended in 100 μl of reconstitution buffer (70% methanol, 10 mM ammonium acetate, pH 8.5). Long chain bases were separated via a reverse-phase C18 column (Uptisphere, 120 Å, 3 μm, 125 × 2 mm; Interchim, France) connected to a QTRAP 6500+ LC-MS/MS System (Sciex, USA). For liquid chromatography, mobile phase A was composed of 10 mM ammonium acetate, 0.2% formic acid in 50% methanol, while 100% methanol was used for mobile phase B. Chromatography was performed at a constant flow rate of 400 μl/min, and the column temperature was held at 50°C throughout. The column was equilibrated with 35% B, followed by a linear increase over 18 min to 65% B, then B was increased to 100% over 1 min and held at 100% for 3 min, reduced to 35% B over 0.5 min, and finally re-equilibrated with 35% B for further 3.5 min. Mass spectra were acquired in multiple-reaction-monitoring mode, and Skyline was used for data integration and analysis (21). For the long chain bases the transitions from precursor m/z to the fragment m/z values corresponding to two dehydrations from the precursor were used in multiple reaction monitoring. 1-deoxy log chain bases were detected from the single dehydration product generated in the ion source with the distinctive pseudo-MS3 fragment corresponding to C2NH6 at m/z 44.1 (22).

### Prediction of DEGS1 structure, docking, and molecular dynamics simulation

The AlphaFold predicted structure AF-O15121-F1 was used for further in silico processing and analysis (23, 24). Reported co- and posttranslational protein modifications were introduced (25, 26, 27). The first methionine residue was removed, the residual residues renumbered, and a myristoyl-group attached to Gly1 utilizing the PDF Manipulator Module of the CHARM-GUI webserver (28). In addition, Ser307 was phosphorylated using the same webserver, and the structural model was optimized utilizing the Protein Preparation Workflow implemented in Maestro (Schrödinger Inc.). In detail, the protonation of the side chains was adjusted to pH 7.0±2.0, H-bonds were assigned and optimized to address any overlapping hydrogen atoms and subsequently the full structure was optimized by running a restrained minimization. A cubic grid with an edge length of 36 Å was positioned at the center of the protein (X: −2, Y: −8, Z: −14) and blind docking of the prepared C16 ceramide ligand was performed using the XP mode in Glide (Schrödinger Inc.). The molecular dynamics simulation was carried out with the Desmond module (integrated in Maestro) employing a TIP3 water model, 150 mM NaCl, a temperature of 310 K and an overall simulation time of 2.5 μsec. To mimic the hydrophobic environment of the ER membrane, DEGS1 with the docked C16 ceramide molecule was positioned in a DPPC model membrane. The position of DEGS1 within the mammalian ER membrane as well as the thickness of the membrane was predicted using the PPM3 webserver (29) utilizing the unmodified AlphaFold model.

### Cell viability assay

HAP1 wild type and HAP1 DEGS1 knockout cells were plated at 10,000 cells per well in black-walled, clear-bottom 96-well microplates and supplemented with increasing concentrations from 0 to 10 μM of SPB 18:1(14Z);O2 (3). The cells were grown in 100 μl growth medium for 48 h, and cell viability was evaluated using a luminescent cell viability assay following the manufacturer's instructions. The cells were cultured in 100 μl growth medium for 48 h, and cell viability was assessed using a luminescent cell viability assay according to the manufacturers’ instructions (CellTiter-Glo, Promega). The luminescence was normalized to the median value of the vehicle treated wells and analyzed using GraphPad Prism version 9.5.1.

## Results

**Fig. 2:**
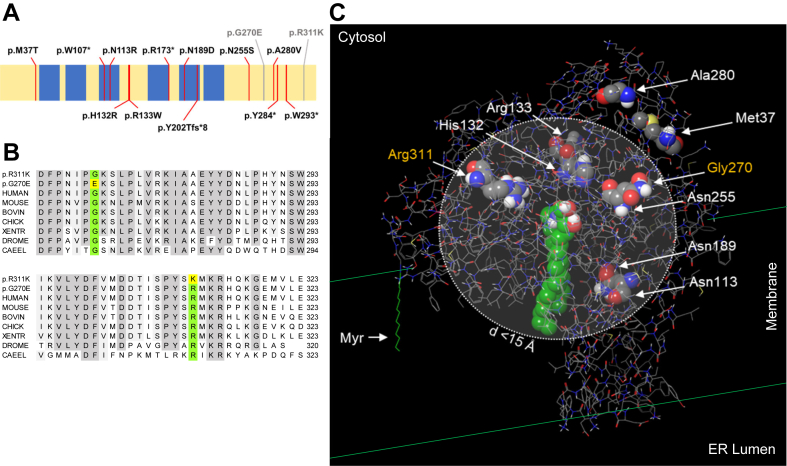
A: Schematic representation of the DEGS1 protein (UniProt ID O15121) displaying known variants of HLD18 (red lines). The new DEGS1 variants are depicted in grey (Patient 1, p.R311K and Patient 2, p.G270E). Putative transmembrane domains are depicted in blue. B: DEGS1 protein alignment of the C-terminal sequence containing the conserved amino acids mutated in the variants p.G270E and p.R311K (green and yellow). C: the location and clustering of single amino acid substitutions leading to HLD18 and the new DEGS1 variants within the predicted protein are shown. The dotted lined circle describes a sphere with a radius of 15 Å. Most variants are confined within the sphere as determined by the distance of the amide nitrogens to the nearest atom of the ceramide ligand, the space-filling model in green. Myr: myristoyl anchor at Gly1.

To date 15 homozygous and compound heterozygous mutations in the *DEGS1* gene have been reported to cause HLD18 (2, 3, 4, 30, 31) (Fig. 2A). The mutations result either in single amino acid substitutions or in truncations of the DEGS1 protein. All previously reported mutations were shown to increase dhSL, thereby altering the dhSL/SL ratio. Here we characterize two *DEGS1* homozygous variants of unknown significance (p.R311K and p.G270E) which were associated with two distinct phenotypes.

**Fig. 3:**
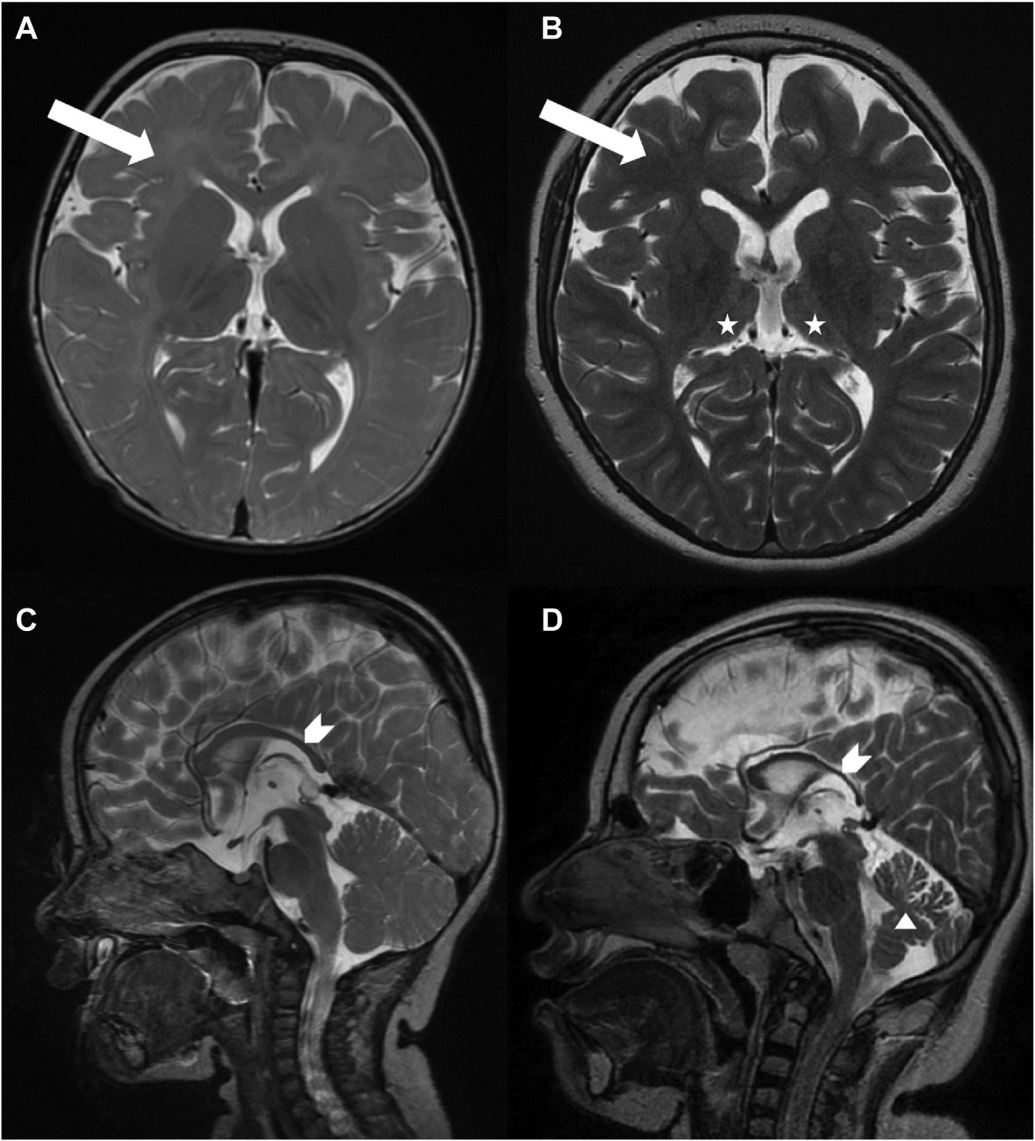
Sequential brain MRI of patient 1: Axial T2-weighted image at the age of 11 months (A) and 11 years (B) respectively showing insufficient myelination (A) (arrow), progressive myelination (B) (arrow) and atrophic thalami (B) (asterisk). Sagittal T2-weighted image at the age of 11 months (C) and 11 years (D) respectively with thinning of the corpus callosum (arrowhead) and cerebellar atrophy (triangle).

The DEGS1 p.R311K (c.932G>A) variant was identified in a 16-year-old girl. She is the first child of healthy consanguineous parents of Sri Lankan origin. Both parents are heterozygous carriers of the VUS. The variant is located in a highly conserved region of DEGS1 (Fig. 2B). The girl was born after an uneventful pregnancy at term, with a birth weight of 2920 g and developed normally until the age of 5–6 months when the failure to thrive began. Tube feeding was initiated at the age of 15 months. Bulbar dysfunction remained unchanged over the years and the patient is mainly fed through a percutaneous endoscopic gastrostomy. Motor development delay was observed in the first year of life; tetraspasticity was noted in the second year of life and became severe during the following years with orthopedic complications (hip dislocation, club foot, and severe scoliosis). She is not able to sit unsupported or to roll over, her head control is limited, and she has flexion contractures of the extremities and a positive Babinski sign. Since the age of 22 months she suffers from pharmacoresistant generalized epilepsy. Her head circumference became microcephalic in the second year of life and has remained 0.5–1 cm below the third percentile to this day. She is alert, maintains eye contact and smiles at speech, but cannot speak. Extensive laboratory investigations and a nerve conduction study were done in the second year of life without abnormalities. Magnetic resonance imaging (MRI) of the brain was done at 11 months, 24 months (not shown), and 11 years. It showed dysmyelination and hypomyelination with some progression in the follow up, a thin corpus callosum, and mild atrophy of the cerebellum and thalami over the years (Fig. 3A–D). The p.R311K variant in *DEGS1* has not yet been described as pathogenic in the literature or mutation databases. The reported minor allele frequency at the gnomAD database is 0.0004015% with no homozygous reports.

The second VUS (p.G270E) was identified in a two-year-old girl born to healthy parents from Somalia. Parents are consanguineous (cousins first order) and heterozygous carriers of the variant. Whole-exome sequencing showed a homozygous variant of uncertain significance in the *DEGS1*-gene (NM_003676.3). The variant changes a highly conserved glycine to glutamic acid (p.G270E) and is not represented in the normal population (e.g. gnomAD database). The girl has four older siblings, all with normal development. She was born after 41 weeks of gestation with a birth weight of 3500 *g*. She was referred to a pediatric neurologist at the age of 7 months, because of delayed psychomotor development. At that age she could not turn her body around, it was hard to get eye contact and she was diagnosed with oculomotor apraxia. At one year of age she was able to sit without support. At 2.5 years of age she was still not able to walk independently. She also has tremor/ataxia in the head, neck and trunk, fine motor deficits and significant speech delay. Metabolic standard screening and genomic microarray were normal. MRI of the brain at 15 months was normal.

### Structural modeling/docking of DEGS1 and location of mutated residues

**Fig. 4:**
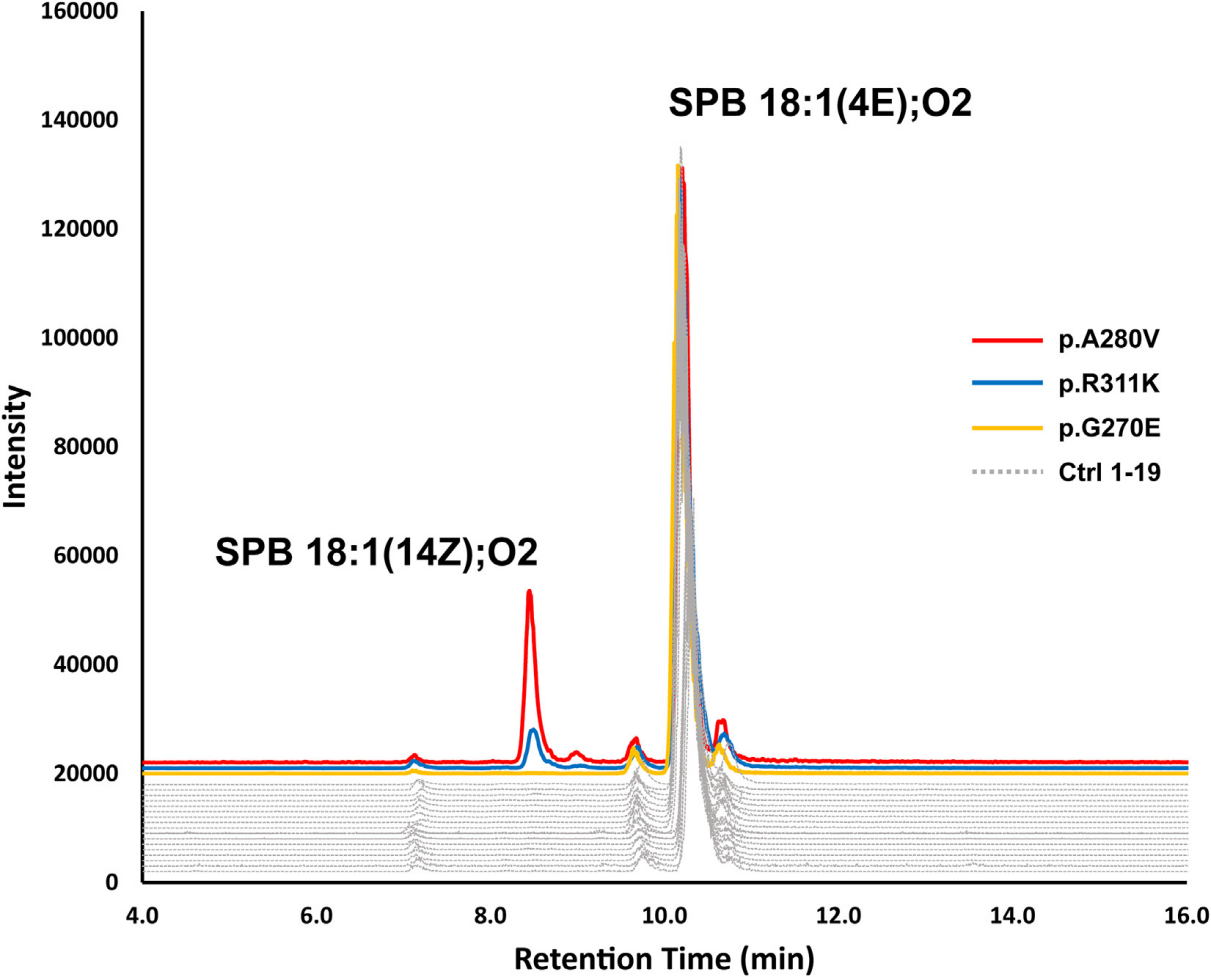
Chromatographic separation of the SPB 18:1(14Z);O2 and SPB 18:1(4E);O2 isomers, resulting from the hydrolysis of complex sphingolipids. SPB 18:1(14Z);O2 is detected in plasma of patients with the p.A280V and p.R311K *DEGS1* variants, but absent in the p.G270E variant and unrelated control plasma obtained from 19 individuals (Ctrl 1–19).

**Fig. 5:**
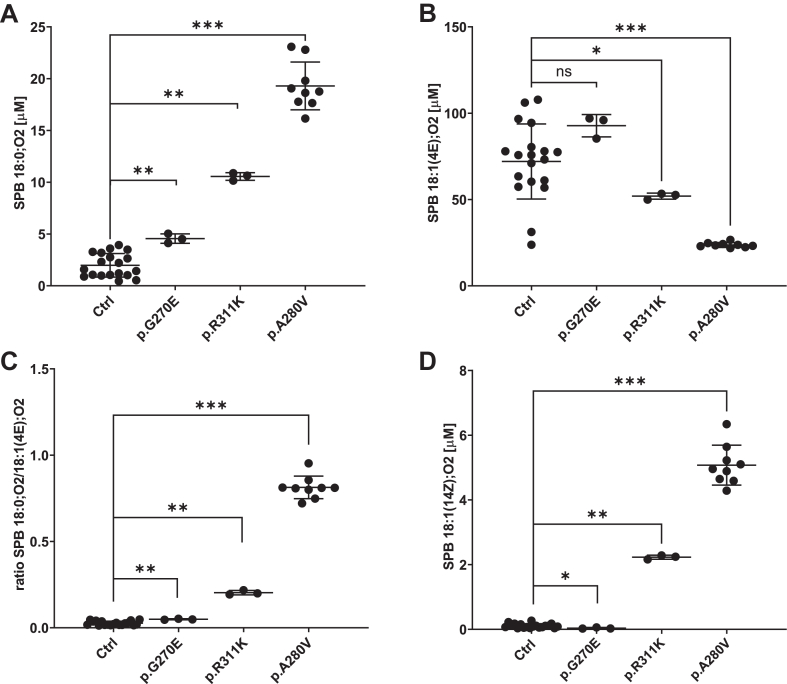
A: The levels of SPB 18:0;O2 is shown with the DEGS1 variants p.G270E, p.R311K and p.A280V, respectively. Ctrl: SPB 18:0;O2 from plasma of 19 unrelated controls. B: The levels of 18:1(4E);O2 detected in plasma of the DEGS1 variants p.G270A, p.R311K and p.A280V, and of 19 unrelated controls. C: The ratio of SPB 18:0;O2/18:1(4E);O2 in plasma of the DEGS1 variants p.G270A, p.R311K and p.A280V, and of 19 unrelated controls. D: The levels of SPB 18:1(14Z);O2 detected in plasma of the DEGS1 variants p.G270A, p.R311K and p.A280V, and of 19 unrelated controls. SPBs shown are the reaction products from the hydrolysis of complex sphingolipids. Data are shown as mean ± SD (error bars), n = 3 for the *DEGS1* variants p.G270E and p.R311K, Average values of 19 unrelated control samples generated from three or four technical replicates each and n = 9 for the *DEGS1* variant p.A280V from 3 biological replicates, Mann-Whitney testing; ∗∗∗*P* < 0.001; ∗∗*P* = 0.001, ∗*P* < 0.05; ns, not significant.

To investigate the potential structural consequences of *DEGS1* mutations on protein conformation and substrate binding, we employed structural modeling and docking simulations. In the absence of an experimentally determined three-dimensional structure of DEGS1, we used a predicted structural model with a high level of confidence for identifying a potential substrate-binding site (supplemental Fig. S1). Based on the cellular location (3, 25, 32), we predicted the arrangement within the ER membrane (supplemental Fig. S2). According to the prediction, the lower, more hydrophobic half of the protein is located within the membrane, whereas the upper, more hydrophilic part is exposed to the cytosol. The myristoyl group at the N-terminal Gly (25, 27) stabilizes the overall orientation of the protein within the membrane. The Cer 18:1(4E);O2/16:0, that we used as a proxy-ligand is guided to the active site via a hydrophobic tunnel and is stabilized via hydrogen bonds between the polar head group of the ceramide and the residues His128/Glu232 (Fig. 2C, supplemental Figs. S2, and S3). Interestingly, while located at the anticipated active site of DEGS1, the hydrocarbon chains of the ceramide mostly remain confined within the membrane. On the other hand, the predicted active site appears to be accessible for potential cytosolic co-factors, such as Fe-ions. A 2500 ns molecular dynamics simulation confirmed the accessibility of the active site for water molecules and indicated a stable DEGS1-ceramide complex despite highly flexible hydrocarbon chains (see supplementary DOI 10.5281/zenodo.8268302). Using this model, we mapped the precise locations of known HLD18 mutations within the DEGS1 protein (Fig. 2C). Most mutations cluster around the anticipated substrate-binding site, while two residues, Ala280 and Met37, are positioned on the periphery of the protein's cytosolic face. The residues at Arg311 and Gly270 both lie within the sphere with a radius of 15 Å as determined by the distance of the amide nitrogens to the nearest atom of the ceramide ligand. It is noteworthy that the substitution of Arg311 with Lys in the p.R311K variant led to a reduction in DEGS1 activity. This reduction could be attributed to a steric hindrance caused by Lys311 during enzyme catalysis. Despite Gly270 being in close proximity to the ceramide ligand, substituting it with Glu in the p.G270E variant did not significantly diminish DEGS1 activity, as revealed by the SPB analysis (Figs. 4 and 5).

### Lipid analysis

DEGS1 deficiency results in increased dhSL levels and an increased dhSL/SL ratio. To determine the metabolic effects of the p.R311K and p.G270E variants on the SL formation, we analyzed the plasma sphingoid base profile of the patients in comparison to healthy controls (Figs. 4 and 5). As dhSL are typically minor and show a mass overlap with several isobaric species (e.g. 18:0;O2/24:1 vs. 18:1;O2/24:0), a direct analysis of dhSLs by mass spectrometry is difficult. We therefore simplified the analysis by subjecting the extracted lipids to chemical hydrolysis prior analysis. During hydrolysis conjugated headgroups and N-acylated fatty acids are removed, resulting in the release of the free SPB backbone. In this way, total dhSLs are converted to sphinganines whereas total SL are converted to sphingosines. Using this approach results in losing the fine structural information of the various complex sphingolipids. Nevertheless, differences in the SPB structures are better resolved than in complex lipid mixtures. In particular, novel SPB forms are detected more likely than it would be the case in complex lipid mixtures.

The SPB analysis of Patient 1, carrying the p.R311K variant showed significantly elevated SPB 18:0;O2 levels compared to healthy controls, whereas total SPB 18:1(4E);O2 levels were reduced (Fig. 5A, B). Consequently the SPB 18:0;O2 to SPB 18:1(4E);O2 ratio was significantly increased, which is in agreement with previous reports of reduced DEGS1 activity (Fig. 5C). In addition, we detected the atypical metabolite SPB 18:1(14Z);O2, which is isomeric to SPB 18:1(4E);O2 but eluted with an about 1.8 min shorter retention time (Fig. 4). In comparison to the previously reported p.A280V variant, the increase in dhSL as well as in SPB 18:1(14Z);O2 was less pronounced for the p.R311K, indicating that the p.R311K mutant retained higher residual activity than p.A280V (Fig. 5A, D).

On the other hand, patient 2 carrying the p.G270E variant showed a modest change in plasma SPB 18:0;O2 levels and minimal increase in the SPB 18:0;O2/18:1(4E);O2 ratio relative to controls (Fig. 5C). Although a statistical significance could be calculated for increased SPB 18:0;O2 levels and a higher SPB 18:0;O2/18:1(4E);O2 ratio, the differences to the control samples are small and do not explain the clinical presentation of Patent 2, carrying the p.G270E variant. Furthermore, the SPB 18:1(14Z);O2 was not detected in the plasma of the p.G270E carrier, and the chromatogram was indistinguishable from the profile of further 19 samples of unrelated healthy controls (Figs. 4 and 5D). Hence, the emergence of SPB 18:1(14Z);O2 in plasma samples could signify a distinctive metabolite exclusively found in HLD18 patients.

**Fig. 6:**
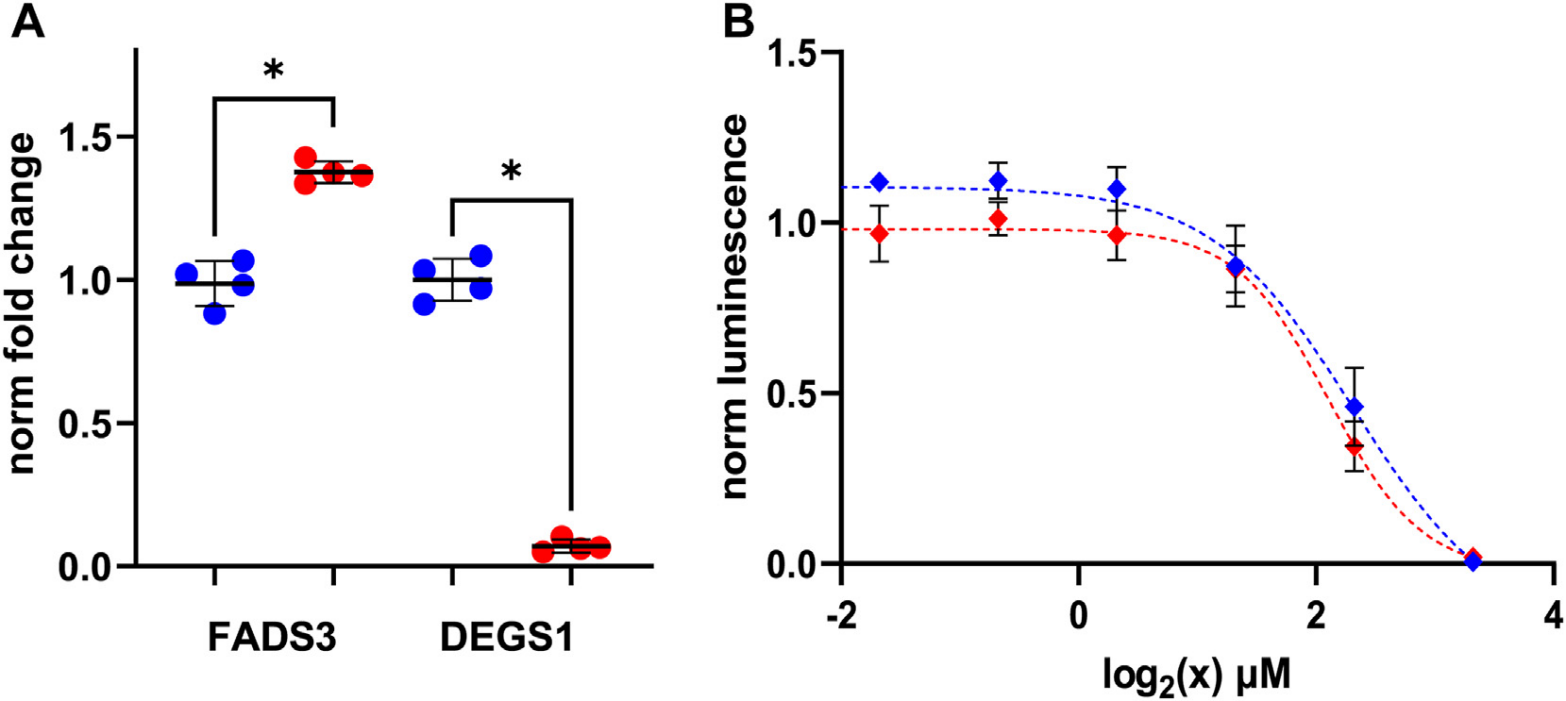
A: *FADS3* expression in HAP1 *DEGS1* knockout cells. mRNA levels of *FADS3* and *DEGS1* were measured by quantitative real-time PCR in wild type (blue dots) and *DEGS1* knockout cells (red dots). Values were normalized to *GAPDH* expression and calculated as fold change relative to wild type cells. Data are shown as median ± SD (error bars), n = 4, Mann-Whitney testing; ∗*P* < 0.05. B: Wild-type HAP1 and *DEGS1* knockout cells were plated in 96-well cell culture plates and supplemented with increasing concentrations of SPB 18:1(14Z);O2 (0–10 μM). Cell viability was assessed after 48 h incubation time, luminescence was normalized to vehicle-treated cells. Data are shown as mean ± SD (error bars), n = 4.

The double bond at Δ14Z is introduced by FADS3, and the desaturase can function either upstream or downstream of DEGS1 in the sphingolipid biosynthetic pathway (20). To explore a potential interplay of DEGS1 deficiency on FADS3 expression, we assessed the expression levels of FADS3 in HAP1 *DEGS1* knockout cells. Interestingly, we detected a significant increase in FADS3 expression in the *DEGS1* knockout cell line, suggesting a regulatory connection with DEGS1 activity (Fig. 6A). Furthermore, when comparing the DEGS1 knockout cell line to wild-type cells, no significant difference in SPB 18:1(14Z);O2-induced cytotoxicity was observed. This suggests that the cytotoxicity of SPB 18:1(14Z);O2 is not dependent on DEGS1 activity (Fig. 6B).

## Discussion

In this study, we reported two patients presenting with two different clinical phenotypes and two new homozygous variants in the *DEGS1*-gene, p.R311K and p.G270E respectively. MRI of patient 1 carrying the p.R311K variant showed the typical findings of hypomyelination with relative preservation of the subcortical white matter, improvement in myelination over time, thin corpus callosum, and atrophy of the cerebellum and the thalami (Fig. 3). Both the clinical symptoms such as severe progressive tetraspasticity, intellectual disability, and epilepsy, as well as the imaging findings, are identical to the published cases with HLD18 (4). The sphingolipid analysis revealed an increase in dhSL in the plasma of patient 1, as well as elevated levels of the atypical metabolite SPB 18:1(14Z);O2 that is consistent with earlier reports of a reduced DEGS1 activity.

The clinical presentation of patient 2 carrying the p.G270E variant is completely different. The clinical symptom of psychomotor delay is unspecific and oculomotor apraxia was not reported with HLD18 in published case series (3, 4). This patient has neither a myelination deficiency nor an abnormal corpus callosum, which would support the diagnosis of HLD18. There is no cerebellar anomaly and no molar tooth sign on MRI, which would support the presumptive clinical diagnosis of Joubert syndrome. Unfortunately, no diagnosis can currently be made for patient 2. In contrast to patient 1, dhSL and the dhSL/SL ratio were normal in the plasma of patient 2 and the metabolite SPB 18:1(14Z);O2 could not be detected. This, in combination with the distinct clinical presentation and the normal brain MRI of patient 2 suggests that the p.G270E variant is benign and likely not the cause for the neurological symptoms.

Based on the structural model of the DEGS1 protein with the mapped disease-causing mutations, one can speculate that the dysfunction of DEGS1 in HLD18 is mediated either by altered substrate binding or by alternative reasons such as protein stability, mislocalization, or varied posttranslational modifications. Indeed, most of the known residues affected by missense mutations, including the residue Arg311, cluster in close spatial proximity to the predicted active site or the binding tunnel (Fig. 2C). Mutations near the substrate-binding site or near the catalytic site are likely to interfere sterically with ceramide binding or the catalytic activity. It is worth emphasizing though that protein structures are highly dynamic, and the structural impact of missense mutations is often difficult to deduce. Two of the HLD18-causing mutations, p.A280V and p.M37T, reside more distant to the putative ceramide binding site, near the protein surface at the cytosolic side. The peripheral location within the protein is unlikely to interfere directly with the substrate binding. This is underpinned by the fact that the Arg311 mutated to Lys (R311K) renders DEGS1 dysfunctional, while Gly270 mutated to Glu (G270E) does not seem to affect protein function. Notwithstanding, mutations at the residues Ala280 and Met37 have severe metabolic consequences, indicating that these residues are involved in alternative cellular events, such as protein-protein interactions or interference with previously described DEGS1 protein phosphorylation or glycosylation (26, 33).

Elevated plasma dhSL and an increase in the dhSL/SL ratio can be used as a biomarker to distinguish benign from disease-causing *DEGS1* variants. However, increased dhSL/SL levels are also found in other conditions unrelated to DEGS1 deficiency. dhSL are generally elevated under conditions of an increased SL *de-novo* synthesis, such as in the presence of SPT gain of function mutations causing ALS (9, 34) or with CerTra syndrome, a neurological syndrome caused by increased activity of the ceramide transporter CERT (35). Therefore, an elevated dhSL/SL ratio alone may not be specific for HLD18. A nongeneric biomarker in this respect would be the atypical metabolite SPB 18:1(14Z);O2. This metabolite is only formed under conditions of DEGS1 deficiency and so far was only detected in the context of a DEGS1-related hypomyelinating phenotype. Therefore, SPB 18:1(14Z);O2 is likely a more specific lipid biomarker for the diagnosis of HLD18.

Whether the increased dhSL/SL ratio or the presence of SPB 18:1(14Z);O2 metabolite or both are related to the underlying pathomechanism is currently not clear. Higher dhSL levels have adverse effects on cellular development. In *Drosophila*, a role of the *DEGS1* orthologue *ifc* in promoting intraluminal vesicle formation was reported to be associated with an increased dhCer/Cer ratio and correlated with reduced exosome secretion (36). In the CNS, exosomes are released from oligodendrocytes and may contribute to the formation of the myelin sheath (37). A reduced exosome secretion due to an altered dhCer/Cer ratio might therefore contribute to the myelination defect observed in HLD18.

The myelin sheath is particularly rich in sphingolipids that are hydroxylated at the second carbon of the N-linked fatty acids (38). This hydroxylation reaction is catalyzed by the fatty acid 2-hydroxylase (FA2H) (39). FA2H deficiency leads to leukodystrophy and hereditary spastic paraplegia in humans (40, 41, 42) and FA2H deficient mice show an initially normal myelin formation but leukodystrophy phenotype at a later age (43). Also, mutations in the alkaline ceramidase 3 (ACER3) cause leukodystrophy (44). ACER3 is a ceramide hydrolase and is involved in the remodeling of the N-acyl chain. This indicated that even minor changes in the structure of certain SL species are associated with myelination defects. This typically requires a specific lipid composition that enables membranes to form the required curvature. The formation of myelin therefore depends on a defined combination of membrane lipids. Increased dhSLs might influence the biophysical properties of the glia membrane. Lipids with a cis double bond such as SPB 18:1(14Z);O2 significantly affect the lateral assembly, fluidity and integrity of the lipid membranes due to the bended alkyl chains, which might disturb the myelination process.

The desaturase FADS3 can introduce the Δ14Z double bond in dihydroceramides species, or downstream of DEGS1 activity to form dienine ceramides (20, 45). FADS3 also inserts the Δ14Z double bond to 1-deoxySLs, non-canonical toxic forms of SLs found in inherited and metabolic disease conditions (20, 46, 47, 48, 49). FADS3 has been associated with conferring cellular detoxification effects, since FADS3 overexpression alleviates 1-deoxy-sphinganine toxicity (46). Based on the data on 1-deoxySL, one would expect increasing FADS3 expression to counteract dihydroceramide induced toxicity in these cells. To address this conjecture we tested the expression levels of FADS3 and the cytotoxicity of SPB 18:1(14Z);O2 in *DEGS1* deficient HAP1 cells. We found increased FADS3 levels in *DEGS1*^*−/−*^ cells, confirming the hypothesis (Fig. 6A). Additionally, we observed a similar sensitivity to SPB 18:1(14Z);O2 supplementation in *DEGS1*^*−/−*^ cells and wild-type cells (Fig. 6B). This indicates that a conversion of SPB 18:1(14Z);O2 to SPB 18:2(4E,14Z);O2 by DEGS1 is not required to counteract cytotoxic effects of SPB 18:1(14Z);O2. The introduction of a Δ14Z double bond into saturated ceramides, like 1-deoxy-sphinganines and dihydroceramides, may be sufficient to mitigate their cellular toxicity. Consequently, FADS3 could represent a promising pharmacological target for intervention in dysregulated dihydroceramide metabolism.

Myelin formation is a complex process involving the wrapping of multiple layers of membranes around an axon. Within this process, gangliosides, in particular GD1a and GT1b, act as ligands for the myelin-associated glycoprotein (MAG, Siglec-4). MAG plays a role in initiating myelination and maintaining the interaction and spacing between myelin and axons by binding (50). It is possible that an increased proportion of dihydrosphingolipids in the ganglioside receptors could disrupt the interaction with MAG. This disruption may occur due to changes in the way the glycosyl-head groups are presented to MAG, potentially leading to dysfunctional myelin formation.

Very recently, DEGS1 was described as being enriched in mitochondria-associated endoplasmic reticulum membrane (MAM) and required for MAM integrity and function (51). A disrupted functionality of MAM and the associated SL dysfunction induced by deficient DEGS1 affected further phospholipid and cholesterol ester biosynthetic pathways, shedding light on the complexity of the underlying pathogenesis of HLD18.

The homozygous deletion of DEGS1 in mice resulted in lethality with incomplete penetrance (52). Surviving homozygous mice showed a highly increased dhCer/Cer ratio associated with severe metabolic and neurologic disturbances. Interestingly, the heterozygous littermates were healthy and showed no neurological phenotype. Instead, the heterozygous mice were resistant to glucocorticoid- and high-fat diet-induced diabetes and showed improved insulin sensitivity. The relationship between reduced DEGS1 activity and improved glucose tolerance was further confirmed in subsequent studies (53, 54). However, other reports indicate that elevated dhCer levels were associated with a higher risk for type-2 diabetes in two cohorts (55). Nevertheless, reducing DEGS1 activity is discussed as a novel therapeutic option for the treatment of cardio-metabolic diseases (56). Given the mutual relationship between reduced DEGS1 activity and myelination defects, caution must be taken with such an approach (45). A partial DEGS1 inhibition might indeed have beneficial metabolic effects but reducing activity to the extent seen in HLD18 patients will very likely cause adverse effects. Whether DEGS1 activity can be tailored by a pharmacological approach that results in a clinically relevant and improved metabolic profile in humans remains to be demonstrated.

## Data Availability

The structure of the DEGS1-ceramide complex as PDB file as well as the trajectory and movies of the molecular dynamics simulation can be accessed via DOI 10.5281/zenodo.8268302. Further data that support the findings of this study are available upon request from the corresponding authors and in the supplemental material of this article.

## Supplemental data

This article contains supplemental data.

## Conflict of interest

The authors declare that they have no known competing financial interests or personal relationships that could have appeared to influence the work reported in this paper.

## References

[1] Wolf N.I., Ffrench-Constant C., van der Knaap M.S. (2021). Hypomyelinating leukodystrophies - unravelling myelin biology. Nat. Rev. Neurol..

[2] Dolgin V., Straussberg R., Xu R., Mileva I., Yogev Y., Khoury R. (2019). DEGS1 variant causes neurological disorder. Eur. J. Hum. Genet..

[3] Karsai G., Kraft F., Haag N., Korenke G.C., Hanisch B., Othman A. (2019). DEGS1-associated aberrant sphingolipid metabolism impairs nervous system function in humans. J. Clin. Invest..

[4] Pant D.C., Dorboz I., Schluter A., Fourcade S., Launay N., Joya J. (2019). Loss of the sphingolipid desaturase DEGS1 causes hypomyelinating leukodystrophy. J. Clin. Invest..

[5] Sabourdy F., Astudillo L., Colacios C., Dubot P., Mrad M., Segui B. (2015). Monogenic neurological disorders of sphingolipid metabolism. Biochim. Biophys. Acta.

[6] Lone M.A., Santos T., Alecu I., Silva L.C., Hornemann T. (2019). 1-Deoxysphingolipids. Biochim. Biophys. Acta Mol. Cell Biol. Lipids.

[7] Boyden L.M., Vincent N.G., Zhou J., Hu R., Craiglow B.G., Bayliss S.J. (2017). Mutations in KDSR cause recessive progressive symmetric erythrokeratoderma. Am. J. Hum. Genet..

[8] Radner F.P., Marrakchi S., Kirchmeier P., Kim G.J., Ribierre F., Kamoun B. (2013). Mutations in CERS3 cause autosomal recessive congenital ichthyosis in humans. PLoS Genet..

[9] Lone M.A., Aaltonen M.J., Zidell A., Pedro H.F., Morales Saute J.A., Mathew S. (2022). SPTLC1 variants associated with ALS produce distinct sphingolipid signatures through impaired interaction with ORMDL proteins. J. Clin. Invest..

[10] Goutman S.A., Guo K., Savelieff M.G., Patterson A., Sakowski S.A., Habra H. (2022). Metabolomics identifies shared lipid pathways in independent amyotrophic lateral sclerosis cohorts. Brain.

[11] Phillips G.R., Saville J.T., Hancock S.E., Brown S.H.J., Jenner A.M., McLean C. (2022). The long and the short of Huntington's disease: how the sphingolipid profile is shifted in the caudate of advanced clinical cases. Brain Commun..

[12] Mill J., Patel V., Okonkwo O., Li L., Raife T. (2022). Erythrocyte sphingolipid species as biomarkers of Alzheimer's disease. J. Pharm. Anal..

[13] Huynh K., Lim W.L.F., Giles C., Jayawardana K.S., Salim A., Mellett N.A. (2020). Concordant peripheral lipidome signatures in two large clinical studies of Alzheimer's disease. Nat. Commun..

[14] Hannun Y.A., Obeid L.M. (2018). Sphingolipids and their metabolism in physiology and disease. Nat. Rev. Mol. Cell Biol..

[15] Lone M.A., Hulsmeier A.J., Saied E.M., Karsai G., Arenz C., von Eckardstein A. (2020). Subunit composition of the mammalian serine-palmitoyltransferase defines the spectrum of straight and methyl-branched long-chain bases. Proc. Natl. Acad. Sci. U. S. A..

[16] Levy M., Futerman A.H. (2010). Mammalian ceramide synthases. IUBMB Life.

[17] Ternes P., Franke S., Zahringer U., Sperling P., Heinz E. (2002). Identification and characterization of a sphingolipid delta 4-desaturase family. J. Biol. Chem..

[18] Yamaji T., Hanada K. (2015). Sphingolipid metabolism and interorganellar transport: localization of sphingolipid enzymes and lipid transfer proteins. Traffic.

[19] Michel C., van Echten-Deckert G., Rother J., Sandhoff K., Wang E., Merrill A.H. (1997). Characterization of ceramide synthesis. A dihydroceramide desaturase introduces the 4,5-trans-double bond of sphingosine at the level of dihydroceramide. J. Biol. Chem..

[20] Jojima K., Kihara A. (2023). Metabolism of sphingadiene and characterization of the sphingadiene-producing enzyme FADS3. Biochim. Biophys. Acta Mol. Cell Biol. Lipids.

[21] Adams K.J., Pratt B., Bose N., Dubois L.G., St. John-Williams L., Perrott K.M. (2020). Skyline for small molecules: a unifying software package for quantitative metabolomics. J. Proteome Res..

[22] Zitomer N.C., Mitchell T., Voss K.A., Bondy G.S., Pruett S.T., Garnier-Amblard E.C. (2009). Ceramide synthase inhibition by fumonisin B1 causes accumulation of 1-deoxysphinganine: a novel category of bioactive 1-deoxysphingoid bases and 1-deoxydihydroceramides biosynthesized by mammalian cell lines and animals. J. Biol. Chem..

[23] Jumper J., Evans R., Pritzel A., Green T., Figurnov M., Ronneberger O. (2021). Highly accurate protein structure prediction with AlphaFold. Nature.

[24] Varadi M., Anyango S., Deshpande M., Nair S., Natassia C., Yordanova G. (2022). AlphaFold Protein Structure Database: massively expanding the structural coverage of protein-sequence space with high-accuracy models. Nucleic Acids Res..

[25] Beauchamp E., Tekpli X., Marteil G., Lagadic-Gossmann D., Legrand P., Rioux V. (2009). N-Myristoylation targets dihydroceramide Delta4-desaturase 1 to mitochondria: partial involvement in the apoptotic effect of myristic acid. Biochimie.

[26] Dephoure N., Zhou C., Villen J., Beausoleil S.A., Bakalarski C.E., Elledge S.J. (2008). A quantitative atlas of mitotic phosphorylation. Proc. Natl. Acad. Sci. U. S. A..

[27] Thinon E., Serwa R.A., Broncel M., Brannigan J.A., Brassat U., Wright M.H. (2014). Global profiling of co- and post-translationally N-myristoylated proteomes in human cells. Nat. Commun..

[28] Jo S., Kim T., Iyer V.G., Im W. (2008). CHARMM-GUI: a web-based graphical user interface for CHARMM. J. Comput. Chem..

[29] Lomize A.L., Todd S.C., Pogozheva I.D. (2022). Spatial arrangement of proteins in planar and curved membranes by PPM 3.0. Protein Sci..

[30] Wong M.S.T., Thomas T., Lim J.Y., Kam S., Teo J.X., Ching J. (2023). DEGS1 -related leukodystrophy: a clinical report and review of literature. Clin. Dysmorphol..

[31] Yan H., Ji H., Kubisiak T., Wu Y., Xiao J., Gu Q. (2021). Genetic analysis of 20 patients with hypomyelinating leukodystrophy by trio-based whole-exome sequencing. J. Hum. Genet..

[32] Cadena D.L., Kurten R.C., Gill G.N. (1997). The product of the MLD gene is a member of the membrane fatty acid desaturase family: overexpression of MLD inhibits EGF receptor biosynthesis. Biochemistry.

[33] Zhang W., Liu T., Dong H., Bai H., Tian F., Shi Z. (2017). Synthesis of a highly azide-reactive and thermosensitive biofunctional reagent for efficient enrichment and large-scale identification of O-GlcNAc proteins by mass spectrometry. Anal. Chem..

[34] Mohassel P., Donkervoort S., Lone M.A., Nalls M., Gable K., Gupta S.D. (2021). Childhood amyotrophic lateral sclerosis caused by excess sphingolipid synthesis. Nat. Med..

[35] Gehin C., Lone M.A., Lee W., Capolupo L., Ho S., Adeyemi A.M. (2023). CERT1 mutations perturb human development by disrupting sphingolipid homeostasis. J. Clin. Invest..

[36] Wu C.Y., Jhang J.G., Lin W.S., Chuang P.H., Lin C.W., Chu L.A. (2021). Dihydroceramide desaturase promotes the formation of intraluminal vesicles and inhibits autophagy to increase exosome production. iScience.

[37] Kramer-Albers E.M., Bretz N., Tenzer S., Winterstein C., Mobius W., Berger H. (2007). Oligodendrocytes secrete exosomes containing major myelin and stress-protective proteins: trophic support for axons?. Proteomics Clin. Appl..

[38] Kishimoto Y. (1986). Phylogenetic development of myelin glycosphingolipids. Chem. Phys. Lipids.

[39] Alderson N.L., Rembiesa B.M., Walla M.D., Bielawska A., Bielawski J., Hama H. (2004). The human FA2H gene encodes a fatty acid 2-hydroxylase. J. Biol. Chem..

[40] Edvardson S., Hama H., Shaag A., Gomori J.M., Berger I., Soffer D. (2008). Mutations in the fatty acid 2-hydroxylase gene are associated with leukodystrophy with spastic paraparesis and dystonia. Am. J. Hum. Genet..

[41] Kruer M.C., Paisan-Ruiz C., Boddaert N., Yoon M.Y., Hama H., Gregory A. (2010). Defective FA2H leads to a novel form of neurodegeneration with brain iron accumulation (NBIA). Ann. Neurol..

[42] Dick K.J., Eckhardt M., Paisan-Ruiz C., Alshehhi A.A., Proukakis C., Sibtain N.A. (2010). Mutation of FA2H underlies a complicated form of hereditary spastic paraplegia (SPG35). Hum. Mutat..

[43] Zoller I., Meixner M., Hartmann D., Bussow H., Meyer R., Gieselmann V. (2008). Absence of 2-hydroxylated sphingolipids is compatible with normal neural development but causes late-onset axon and myelin sheath degeneration. J. Neurosci..

[44] Edvardson S., Yi J.K., Jalas C., Xu R., Webb B.D., Snider J. (2016). Deficiency of the alkaline ceramidase ACER3 manifests in early childhood by progressive leukodystrophy. J. Med. Genet..

[45] Tzou F.Y., Hornemann T., Yeh J.Y., Huang S.Y. (2023). The pathophysiological role of dihydroceramide desaturase in the nervous system. Prog. Lipid Res..

[46] Karsai G., Lone M., Kutalik Z., Brenna J.T., Li H., Pan D. (2020). FADS3 is a Delta14Z sphingoid base desaturase that contributes to gender differences in the human plasma sphingolipidome. J. Biol. Chem..

[47] Gantner M.L., Eade K., Wallace M., Handzlik M.K., Fallon R., Trombley J. (2019). Serine and lipid metabolism in macular disease and peripheral neuropathy. N. Engl. J. Med..

[48] Othman A., Rutti M.F., Ernst D., Saely C.H., Rein P., Drexel H. (2012). Plasma deoxysphingolipids: a novel class of biomarkers for the metabolic syndrome?. Diabetologia.

[49] Penno A., Reilly M.M., Houlden H., Laura M., Rentsch K., Niederkofler V. (2010). Hereditary sensory neuropathy type 1 is caused by the accumulation of two neurotoxic sphingolipids. J. Biol. Chem..

[50] Schnaar R.L. (2023). Gangliosides as Siglec ligands. Glycoconj J..

[51] Planas-Serra L., Launay N., Goicoechea L., Heron B., Jou C., Julia-Palacios N. (2023). Sphingolipid desaturase DEGS1 is essential for mitochondria-associated membrane integrity. J. Clin. Invest..

[52] Holland W.L., Brozinick J.T., Wang L.P., Hawkins E.D., Sargent K.M., Liu Y. (2007). Inhibition of ceramide synthesis ameliorates glucocorticoid-, saturated-fat-, and obesity-induced insulin resistance. Cell Metab..

[53] Bikman B.T., Summers S.A. (2011). Ceramides as modulators of cellular and whole-body metabolism. J. Clin. Invest..

[54] Chaurasia B., Tippetts T.S., Mayoral Monibas R., Liu J., Li Y., Wang L. (2019). Targeting a ceramide double bond improves insulin resistance and hepatic steatosis. Science.

[55] Wigger L., Cruciani-Guglielmacci C., Nicolas A., Denom J., Fernandez N., Fumeron F. (2017). Plasma dihydroceramides are diabetes susceptibility biomarker candidates in mice and humans. Cell Rep..

[56] Blitzer J.T., Wang L., Summers S.A. (2020). DES1: a key driver of lipotoxicity in metabolic disease. DNA Cell Biol..

