# Supplementary material for: The atypical sphingolipid SPB 18:1(14Z);O2 is a biomarker for DEGS1 related hypomyelinating leukodystrophy
Source: J Lipid Res. 2023 Oct 27;64(12):100464. doi: 10.1016/j.jlr.2023.100464 (PMC10696257; doi:10.1016/j.jlr.2023.100464)
Supplement: Supplemental data [file mmc1.pdf]

The atypical sphingolipid SPB 18:1(14Z);O2 is a biomarker for *DEGS1* related hypomyelinating leukodystrophy

Andreas J. Hülsmeier[1,2], Sandra P Toelle[3], Peter Bellstedt[1], Christian Wentzel[4], Angela Bahr[5], Konstantinos Kolokotronis[5] and Thorsten Hornemann[1,2]

[1]Institute of Clinical Chemistry, University Hospital Zurich, University of Zurich, Zurich, Switzerland
[3]Department of Pediatric Neurology, University Children's Hospital, Zurich, University of Zurich, Zurich, Switzerland
[4]Department of Women's and Children's Health, Pediatric oncological and neurological research, Uppsala University, Uppsala, Sweden
[5]Institute of Medical Genetics, University of Zurich, Schlieren, Zurich, Switzerland
[2]Corresponding authors: andreas.huelsmeier@uzh.ch, thorsten.hornemann@usz.ch

Suppl. Fig. 1:

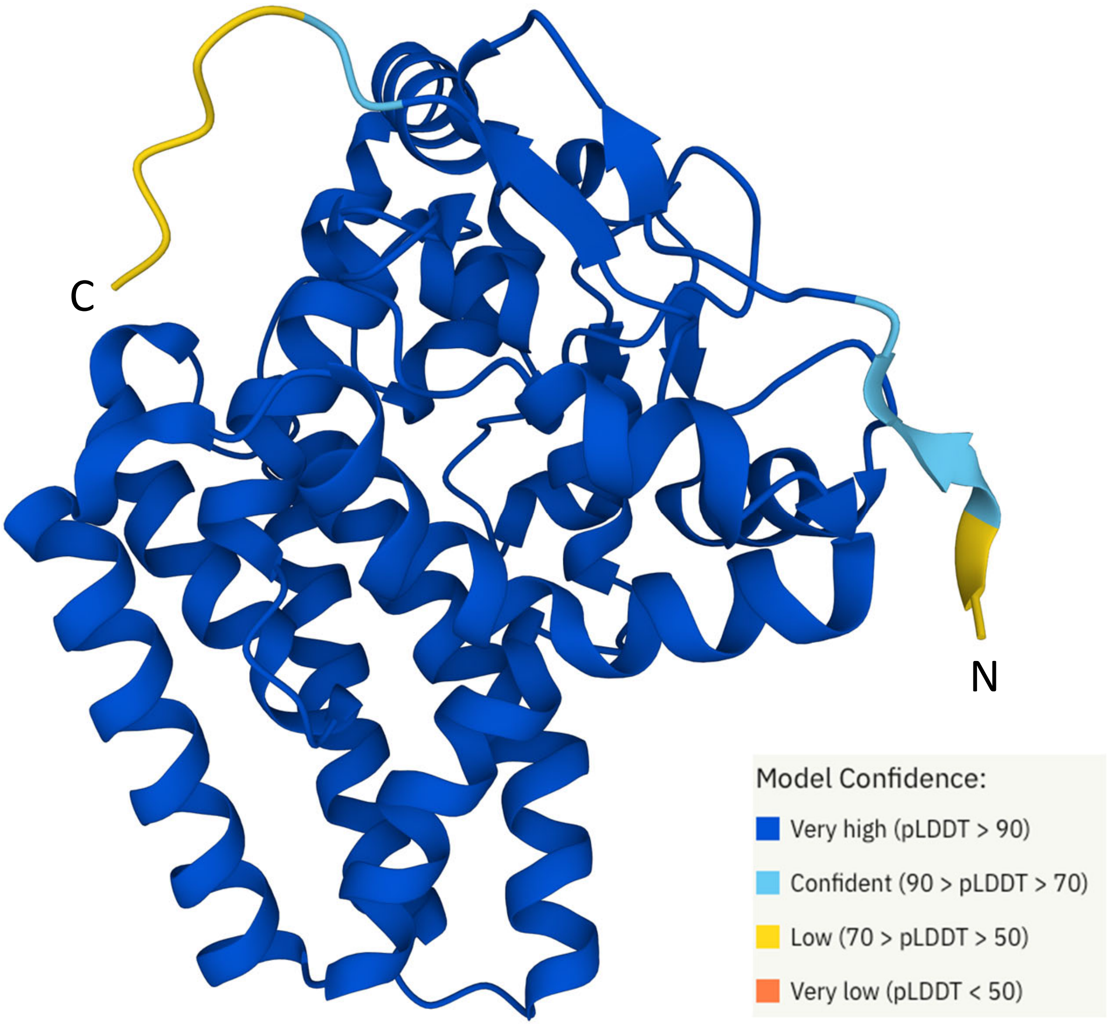

Suppl. Fig. 1: Unprocessed model of DEGS1 as obtained from AlphaFold. Color coding represent the per-residue confidence score (pLDDT) suggesting a high model confidence of the overall structure.

Suppl. Fig. 2:

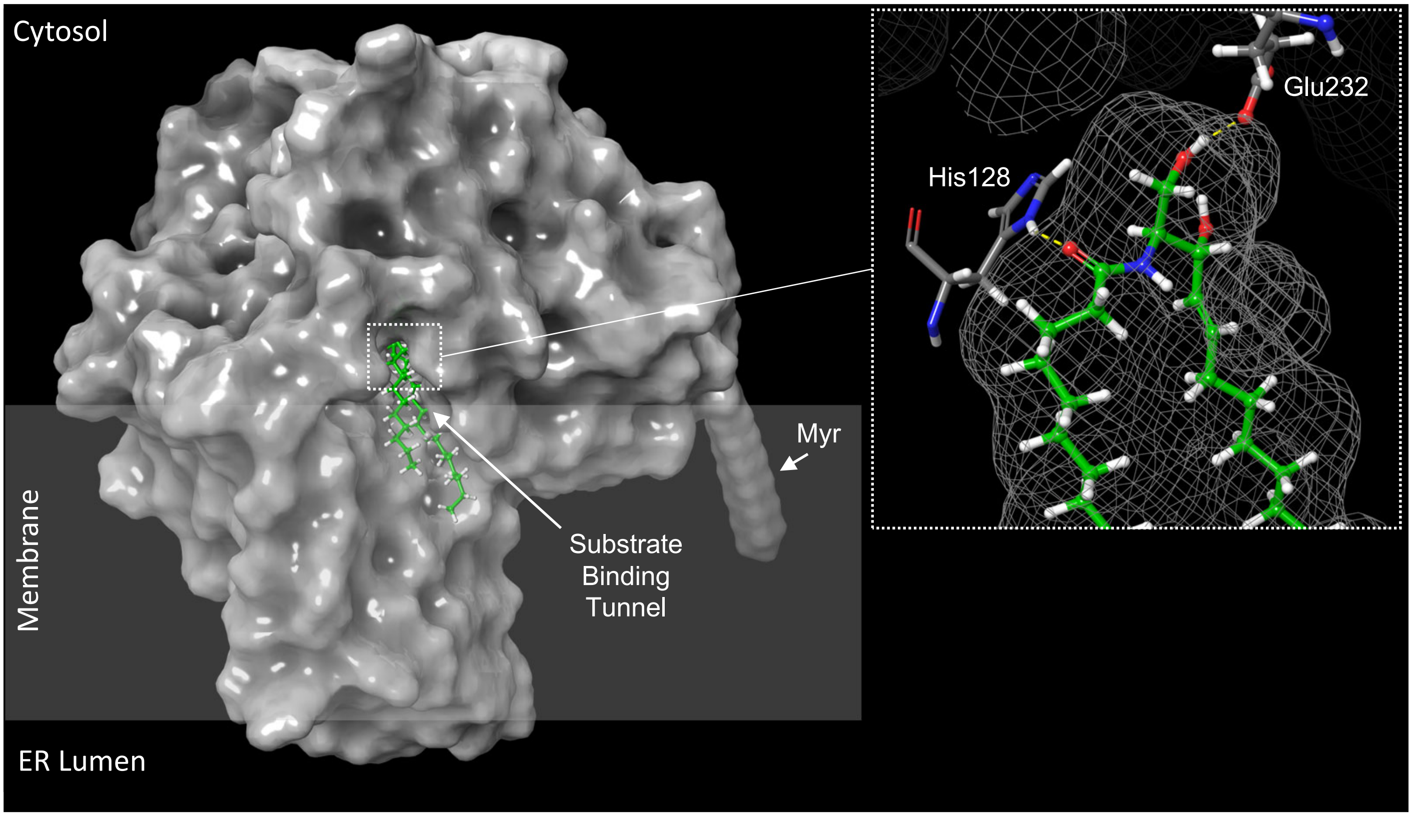

Suppl. Fig. 2: Predicted DEGS1 structure including the binding site of Cer 18:1(4E);O2/16:0 as obtained from docking experiments. For clarity, carbons are depicted in green. The polar head group of the ceramide is coordinated at the end of the binding tunnel via predicted hydrogen bond interactions formed from His128 to the carbonyl group and from Glu232 to the hydroxy group at C1. The hydrocarbon chains remain confined within the membrane. Myr: myristoyl anchor at Gly1.

Suppl. Fig. 3:

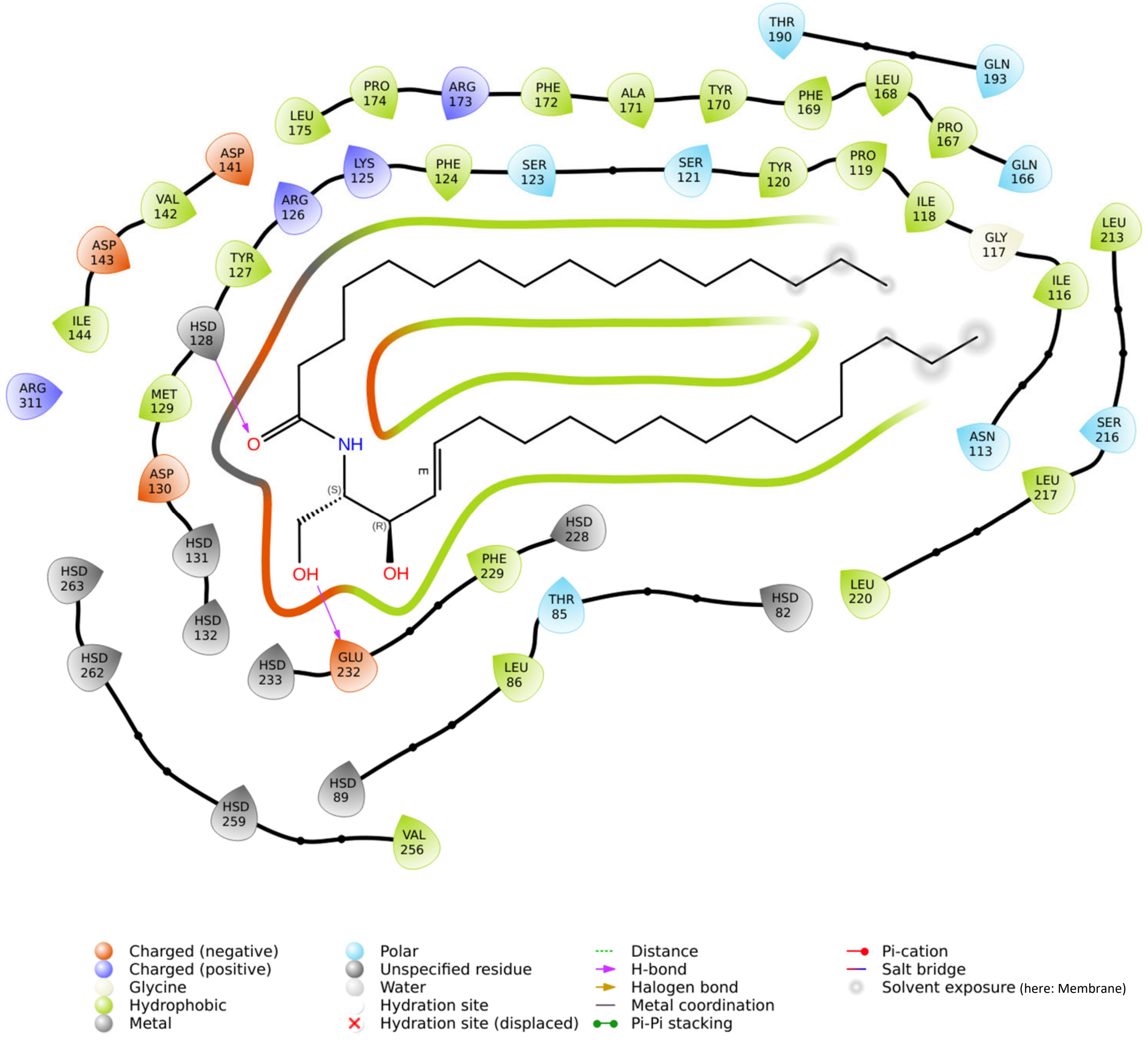

Suppl. Fig. 3: Ligand interaction diagram of the predicted DEGS1 model docked to Cer 18:1(4E);O2/16:0. The two hydrogen bond interactions that arrange and stabilize the polar head group and the hydrophobic environment that build up the binding tunnel (green lines) are depicted.
